# Laser capture microdissection transcriptome (LCM RNA-seq) reveals *BcDFR* is a key gene in anthocyanin synthesis of non-heading Chinese cabbage

**DOI:** 10.1186/s12864-024-10341-y

**Published:** 2024-04-29

**Authors:** Qian Zhou, Xinfeng Xu, Mengjie Li, Xiaoxue Yang, Meiyun Wang, Ying Li, Xilin Hou, Tongkun Liu

**Affiliations:** 1 State Key Laboratory of Crop Genetics & Germplasm Enhancement, Key Laboratory of Biology and Genetic Improvement of Horticultural Crops (East China), Ministry of Agriculture and Rural Affairs of China, Engineering Research Center of Germplasm Enhancement and Utilization of Horticultural Crops, Ministry of Education of China, Nanjing Agricultural University, Nanjing, 210095 China; 2 Nanjing Suman Plasma Engineering Research Institute, Nanjing Agricultural University, Nanjing, 210095 China

**Keywords:** LCM RNA-Seq, Anthocyanin, *BcDFR*, Non-heading Chinese cabbage

## Abstract

**Background:**

Purple non-heading Chinese cabbage [*Brassica campestris* (syn. *Brassica rapa*) ssp. *chinensis*] has become popular because of its richness in anthocyanin. However, anthocyanin only accumulates in the upper epidermis of leaves. Further studies are needed to investigate the molecular mechanisms underlying the specific accumulation of it.

**Results:**

In this study, we used the laser capture frozen section method (LCM) to divide purple (ZBC) and green (LBC) non-heading Chinese cabbage leaves into upper and lower epidermis parts (Pup represents the purple upper epidermis, Plow represents the purple lower epidermis, Gup represents the green upper epidermis, Glow represents the green lower epidermis). Through transcriptome sequencing, we found that the DIHYDROFLAVONOL 4-REDUCTASE-encoding gene *BcDFR*, is strongly expressed in Pup but hardly in others (Plow, Gup, Glow). Further, a deletion and insertion in the promoter of *BcDFR* in LBC were found, which may interfere with *BcDFR* expression. Subsequent analysis of gene structure and conserved structural domains showed that *BcDFR* is highly conserved in *Brassica species*. The predicted protein-protein interaction network of *BcDFR* suggests that it interacts with almost all functional proteins in the anthocyanin biosynthesis pathway. Finally, the results of the tobacco transient expression also demonstrated that *BcDFR* promotes the synthesis and accumulation of anthocyanin.

**Conclusions:**

*BcDFR* is specifically highly expressed on the upper epidermis of purple non-heading Chinese cabbage leaves and regulates anthocyanin biosynthesis and accumulation. Our study provides new insights into the functional analysis and transcriptional regulatory network of anthocyanin-related genes in purple non-heading Chinese cabbage.

**Supplementary Information:**

The online version contains supplementary material available at 10.1186/s12864-024-10341-y.

## Introduction

Anthocyanins are natural, water-soluble flavonoid pigments that provide a variety of colors to plant leaves, petals, and fruit. Bright colors help plants attract animals for pollination. It also has antioxidant activity and enhances the antioxidant capacity of plants. Studies have shown that anthocyanins have pharmacological effects such as anti-aging, anti-allergy, cardiovascular protection, anti-inflammatory, obesity prevention, antioxidant, free radical scavenging, vision improvement, and cancer prevention [1]. Anthocyanin-rich fruits and vegetables have also become more attractive because of their beneficial effects on human health [2].

The pathway of anthocyanin biosynthesis has been well characterized in numerous species, including *Arabidopsis*, maize (*Zea mays*), petunia, and snapdragon [3, 4]. The anthocyanin biosynthetic pathways include the phenylpropanoid pathway, early biosynthesis stage and later biosynthesis stage. Therefore, the genes involved are also divided into two subgroups: early biosynthetic genes (EBGs) and late biosynthetic genes (LBGs) [5]. Firstly, the primary phenylalanine pathway includes phenylalanine ammonia lyase (PAL), cinnamate 4-hydroxylase (C4H) and 4-coumarate: CoA ligase (4CL), which provide precursor substrates P-coumaroyl-CoA for flavonoid synthesis [6]. Secondly, the early biosynthetic pathway, includes chalcone synthase (CHS), chalcone isomerase (CHI), flavanone 3-hydroxylase (F3H), flavanone 3′-hydroxylase (F3’H) and flavanol synthase (FLS), which provide precursor substrates for the synthesis of flavanol and anthocyanin [5]. CHS catalyzes the synthesis of naringenin chalcones from P-coumaroyl-CoA, followed by isomerization to colorless chalcones in the presence of CHI. The chalcones are further catalyzed by flavanone hydroxylase (F3′5′H; F3′H; F3H) to give colorless dihydroflavonols [7]. Thirdly, the late biosynthetic pathway includes dihydroflavonol 4-reductase (DFR), anthocyanin synthase (ANS), UDP-glucosyltransferase (UGT) and acyltransferase (AT), which complete the biosynthesis and modification of anthocyanins [3]. The reduction of dihydroflavonols to colorless anthocyanins catalyzed by DFR, followed by oxygenation by ANS to colored anthocyanin glycosides, and ultimately by UFGT and AT, catalyzed the formation of anthocyanin glycosides with different colors [7]. FLS and DFR compete to produce either flavanols or anthocyanins, respectively [6]. Moreover, anthocyanin biosynthesis is regulated by a variety of transcription factors (TFs). MYB, bHLH and WD40 always form a transcriptional activation complex (MBW) to co-regulate the expression of the genes involved in anthocyanin biosynthesis [8].

Many genes involved in anthocyanin biosynthesis in plants have already been cloned and studied. The discovery of *COLORED1* (*C1*), the first anthocyanin-related R3R3-MYB gene found in maize (*Zea mays*), has revealed the intricate regulatory mechanisms behind anthocyanin production [9]. This pioneering research reveals the secrets of nature’s vibrant palette. Subsequently, *AtPAP1* (*AtMYB75*), the first anthocyanin-related R2R3-MYB transcription factor in *Arabidopsis*, was identified. Interestingly, AtMYB75 together with the bHLH proteins AtGL3, AtEGL3, and AtTT8, as well as the WDR protein TTG1, it regulates anthocyanin accumulation by forming the MYB-bHLH-WDR (MBW) complex [10]. Vibrant hues are indispensable for showcasing fruits. In apples, the MdMYB1/MYBA transcription factor was found to be associated with apple peel color, while MdMYB10 is the key player responsible for the production of anthocyanin in the fruit [11–13]. *PpMYB10* promotes anthocyanin accumulation in pear by regulating genes encoding anthocyanin pathway enzymes [14]. In tomatoes, the colorful fruits are also attributed to *LeANT1* and *LeAN2* [15, 16]. In rice, three genes have been revealed as essential regulators of leaf anthocyanin synthesis, including *OsC1*, *OsRb* and *OsDFR* [17].

Brassica contains many vegetables rich in anthocyanins. In Brassica species, many studies have been devoted to the discovery of genes associated with anthocyanin accumulation and their functional analysis. In rapid-cycling *B. rapa*, an anthocyaninless (*anl*) locus, which inhibits anthocyanin synthesis, had been mapped to R9 [18]. Two *AtEGL3* homologs, *BrEGL3.1* and *BrEGL3.2*, had been identified on chromosome A09, encoding bHLH transcription factors, which are candidate genes associated with anthocyanin accumulation in zicaitai (*B. rapa* L. ssp. c*hinensis* var. *purpurea*) [19]. The *BoDRF* gene, which regulates the synthesis of anthocyanins in the stems of Chinese kale (*Brassica oleracea var. alboglabra*), had been fine-mapped to chromosome C09 [20]. *BrPur*, a single dominant gene controlling endophytic leaf anthocyanin accumulation in Chinese cabbage, had been mapped to linkage group A07 [21]. In *B. napus*, the *BnAPR2* gene was mapped on chromosome A03 as a candidate for regulating leaf purple color [22]. Also, many genes related to anthocyanin accumulation have been cloned. For example, low temperature treatment induced the expression of *BoTT8* and *BoMYB2/BoPAP1* and regulated the accumulation of anthocyanins in the purple kale [23]. In purple mustard, up-regulation of anthocyanin biosynthesis genes induced the expression of *BjTT8*, which promoted anthocyanin synthesis [24]. Meanwhile, the purple cabbage MYB homolog *BoMYBL2–1* was predicted to be a negative regulator of anthocyanin synthesis [25]. In all, these studies provide insight into the coloration mechanisms of Brassica species. However, the discovery and functional analysis of anthocyanin-related genes in Brassica vegetables still need to be further explored.

Non-heading Chinese cabbage [*Brassica campestris* (syn. *Brassica rapa*) ssp. *chinensis*] is an important leafy vegetable cultivated in Asia [26]. The leaf color is generally green. In recent years, purple non-heading Chinese cabbages have received more attention for their vibrant colors and high content of anthocyanins. Interestingly, anthocyanin in the purple variety is mainly accumulated in the upper epidermis and veins of the leaves, with almost no anthocyanin in the lower epidermis. In this study, two varieties (purple ZBC and green LBC) of non-heading Chinese cabbage were used to investigate the key genes involved in anthocyanin regulatory pathway. The upper and lower epidermis of leaves were collected from both varieties at the same growth period using a laser capture frozen section method (LCM) to perform RNA-seq. Finally, we found that *BcDFR* may be a key gene regulating the specific accumulation of anthocyanin on the upper epidermis of purple non-heading Chinese cabbage leaves. Our study provides new insights into transcriptional regulatory network of anthocyanin-related genes in purple non-heading Chinese cabbage.

## Materials and methods

### Plant materials

Two varieties of non-heading Chinese cabbage were used in our study, purple ZBC and green LBC. F1 is a cross between ZBC and LBC. Seeds were placed in pots with substrate (soil matrix and vermiculite 1:1) and grown in a climate chamber with long-day conditions (16 h of light, 22 °C/8 h of darkness 18 °C). In addition, robust ZBC at the 7-leaf stage were selected for low-temperature treatment (4 °C, 30 days). All materials used in this study were provided by Nanjing Agricultural University’s Chinese Cabbage System Biology Laboratory.

### Total anthocyanin analysis

For anthocyanin measurement, 0.1 g of fresh leaf tissue was collected. The leaves were thoroughly crushed in liquid nitrogen, followed by the addition of 2 ml of acidified methanol (99 CH3OH:1HCl, v/v) and extraction for 24 hours at low temperature in the dark. Then 12,000×g centrifugation was performed for 5 min, and the absorbance of supernatants was determined using a UV-visible spectrophotometer from optical density (OD) at 530 and 650 nm. Total anthocyanin content (mg/g) = (OD530–0.25 × OD650) × V/0.0462/M. Where V was total volume of extract, M was leaf mass [27].

### Laser-capture microdissection

Tissue specimens were collected from the purple and green leaves. Samples were wrapped using (OCT, Sakura, USA) compound and subsequently cut samples into 20 μm sections using a cryomicrotome (Leica CM3050S, Germany) at − 20 °C. The tissue sections were then uniformly adhered to PEN membrane slides (Leica). Segmentation of the tissue sections into upper and lower parts using an automated laser microdissection system (Leica, LMD7000) [28].

### RNA extraction and transcriptome analysis

Total RNA was extracted from LCM leaf samples using the PicoPureTM RNA Isolation Kit (Thermo Fisher Scientific). Total RNA quantity and purity were analyzed using an Agilent 2100 Bioanalyzer and NanoPhotometer spectrophotometer (Agilent, CA, USA). High quality RNA samples with RIN > 7.0 were used for sequencing library construction. mRNA was purified from total RNA (5 μg) using Dynabeads Oligo (dT) (Thermo Fisher, CA, USA). mRNA was subsequently fragmented into short pieces and reverse transcribed to cDNA using SuperScript™II reverse transcriptase (Invitrogen, cat. 1,896,649, USA). The average insert size for the final cDNA libraries was 300 ± 50 bp. Finally, 2 × 150 bp paired-end sequencing (PE150) was performed on an Illumina Hiseq2000 Truseq SBS Kit v3-HS (200 cycles) following the vendor’s recommended protocol. After performing quality filtering on the raw sequencing data, we ended up with clean data of 461,721,426 reads. The cultivar NHCC001 v1.0 genome (https://www.tbirs.cn/NHCCDB/Genome.jsp) was used as the reference genome. Differentially expressed genes (DEGs) were identified using DEseq2 with screening criteria of FDR < 0.05, |log2FoldChange| ≥ 1.00. All DEGs were annotated and classified using the GO (http://www.geneontology.org/) and the KEGG (http://www.kegg.jp/kegg/).

### Quantitative real-time PCR

cDNA was synthesized using Hifair® AdvanceFast One step RT gDNA Digestion SuperMix for qPCR (Yessen Biotech Co. Ltd., China). Subsequently, qPCR analyses were performed on the StepOnePlus system (Applied Biosystems, USA) using Hieff®qPCR SYBR Green Master Mix (High Rox) (Yessen Biotech, Co. Ltd., China) according to the instructions. The relative expression of genes was analyzed using the 2^−∆∆CT^ method, and the expression of *BcPP2A* (*BraC07g034860.1*) and *BcActin* (*BraC05g034900.1*) was used as a reference. All primers used for qRT-PCR were listed in Supplementary Data Table S1.

### Cloning and analysis of BcDFR

We amplified the *BcDFR* sequences of ZBC and LBC lines and sequenced them by Tsingke Biotechnology Company (Nanjing, China). Primers used for cloning were shown in Supplementary Data Table S1. The MEME (https://meme-suite.org/meme/) was used to analyze the conserved motif sequence of the *DFR* genes. *DFR* promoter sequences for Arabidopsis and six Brassica species were obtained from BRAD (http://brassicadb.cn/#/). Cis-elements were predicted using the PlantCARE database (https://bioinformatics.psb.ugent.be/webtools/plantcare/html/). STRING (https://cn.string-db.org/) was used to predict BcDFR protein interaction network. Anthocyanin-related DEGs specifically expressed on the upper surface of leaves constructed the interaction network of BcDFR. We set the minimum required score for the interaction to medium confidence (0.40).

### BcDFR transient expression assay

The *BcDFR* gene was cloned into the pRI101 vector. A *tumefaciens strain* GV3101 containing *35S:BcDFR* construct was independently inoculated into LBC cotyledons for transient expression [29].

### Statistical analysis

SPSS software was used for the statistical analyses. All data are presented as the mean ± standard deviation of three biological replicates. One-way ANOVA at 0.05 significance level was used to evaluate differences between samples.

## Results

### Phenotypic analysis of two non-heading cabbage varieties

**Fig. 1:**
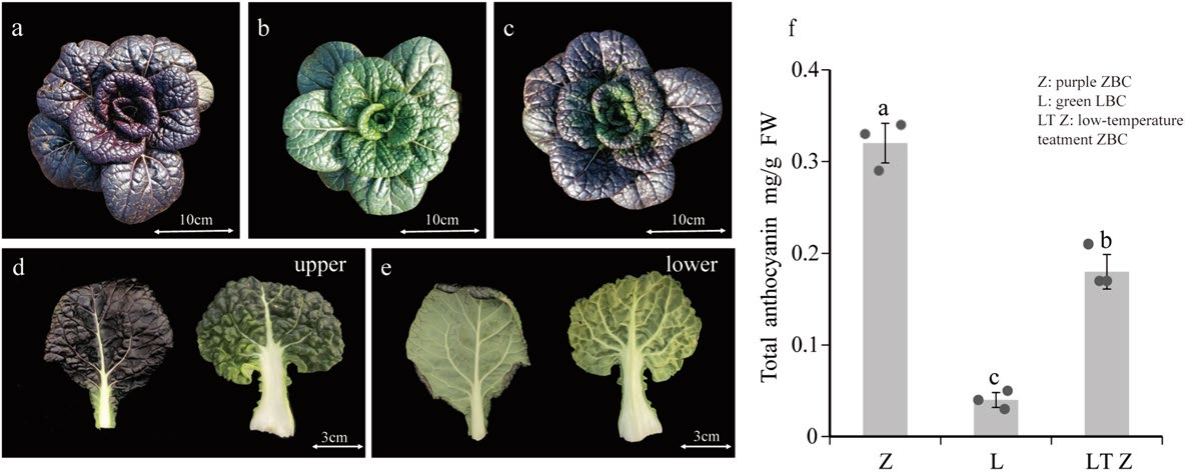
Phenotype and anthocyanin content of ZBC and LBC. **a**-**b** Phenotypes of ZBC and LBC. (c) Phenotype of ZBC after low temperature treatment. **d**-**e** Phenotypes of upper (d) and lower (e) epidermis of leaves. **f** Total anthocyanin content of ZBC, LBC and LTZ. Bar charts represent mean values and scatter plots represent individual data values. Z, ZBC; L, LBC; LTZ, ZBC after low temperature treatment. The data are from three independently repeated experiments. Error bars represent ± SD. Different letters indicate significant differences (*p* < 0.05)

The purple and green non-heading Chinese cabbage varieties (ZBC and LBC) were used in this study (Fig.1a, b). Total anthocyanin content of the two varieties was measured and was 0.32 mg/g for ZBC while only 0.04 mg/g for LBC (Fig.1f). As the leaves developed, anthocyanins accumulated mainly on the upper leaf surface of ZBC, with almost no anthocyanin accumulation in the lower epidermis (Fig. 1)de. Previous studies have reported that anthocyanin accumulation was associated with low temperature [30]. To investigate whether anthocyanin accumulation in non-heading Chinese cabbage responds to low temperature, we treated the purple ZBC with 4 °C for 30 days. Compared with the control, ZBC after low temperature treatment showed internal leaf discoloration (Fig.1c). In addition, the total anthocyanin content also decreased by 43.75% compared with control, only 0.18 mg/g (Fig.1f). In short, the upper epidermis of the purple ZBC accumulates a large amount of anthocyanin and is affected by low temperature.

### Laser-capture microdissection and RNA-Seq

**Fig. 2:**
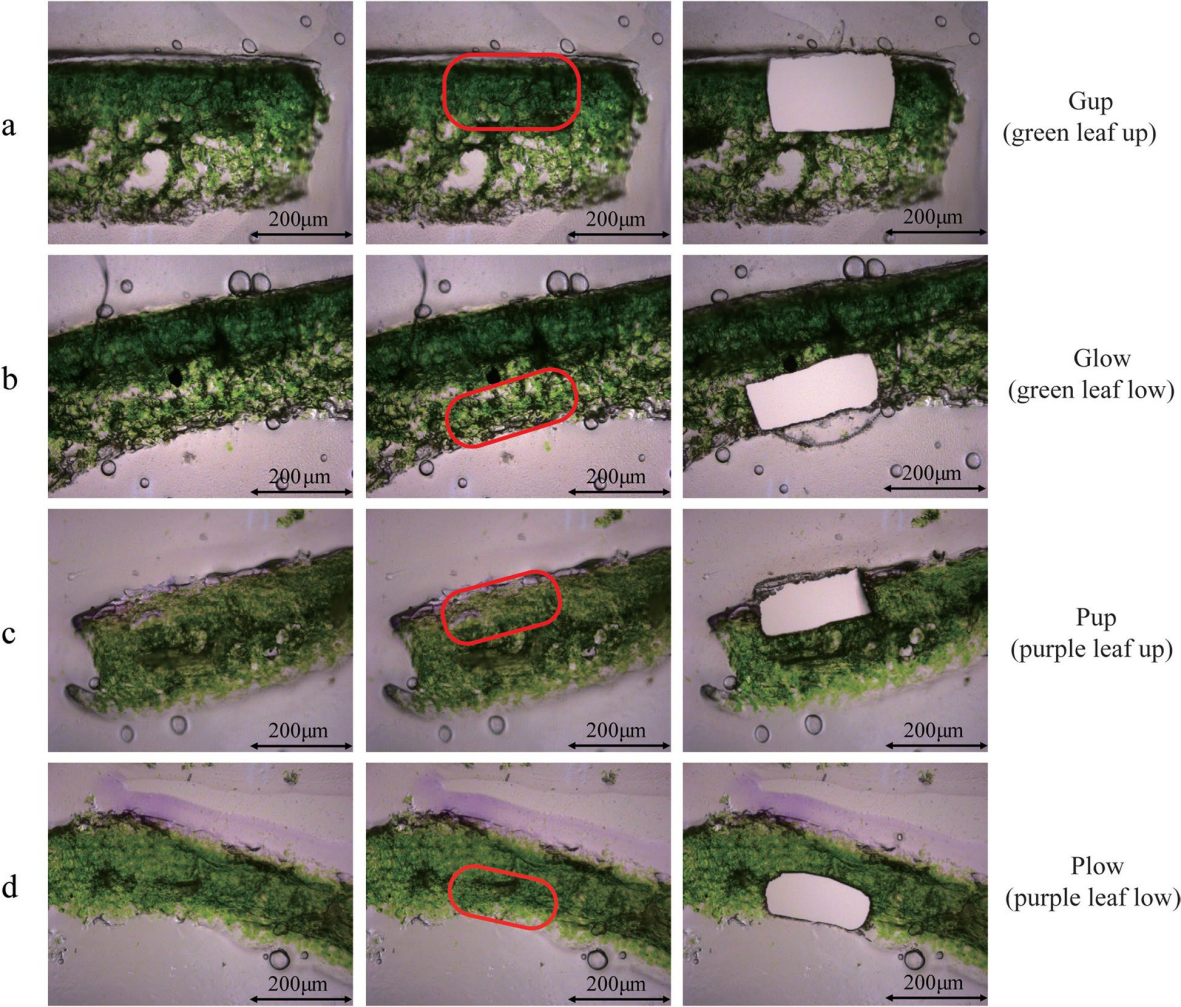
Samples harvested by Laser capture microdissection method. **a** Gup, green LBC leaf upper epidermis. **b** Glow, green LBC leaf lower epidermis. **c** Pup, purple ZBC leaf upper epidermis. **d** Plow, purple ZBC leaf lower epidermis

To investigate genes specifically expressed on the upper epidermis of purple leaves, upper and lower epidermis tissues of leaves from the same growth period were collected from ZBC using a laser capture microdissection frozen section method [28]. Meanwhile, to avoid the difference other than anthocyanins between the upper and lower epidermis, the green LBC was used as a control. Hence, four sequencing libraries were constructed with three replicates. Gup, LBC leaf upper epidermis (Fig.2a), Glow, LBC leaf lower epidermis (Fig.2b), Pup, ZBC leaf upper epidermis (Fig.2c), Plow, ZBC leaf lower epidermis (Fig.2d). Four samples were sequenced by Illumina technology and aligned to the reference genome. After removing adapters and unknown or poor-quality reads, 40,441,648-44,345,740; 41,387,390-9,937,566; 38,134,220-44,624,234 and 39,410,282-42,125,688 clean reads remained in Pup, Plow, Gup and Glow respectively. The Q30 score for each sample was 93.40–94.29% and the mean GC content was 48.44% (Table S2).

**Fig. 3:**
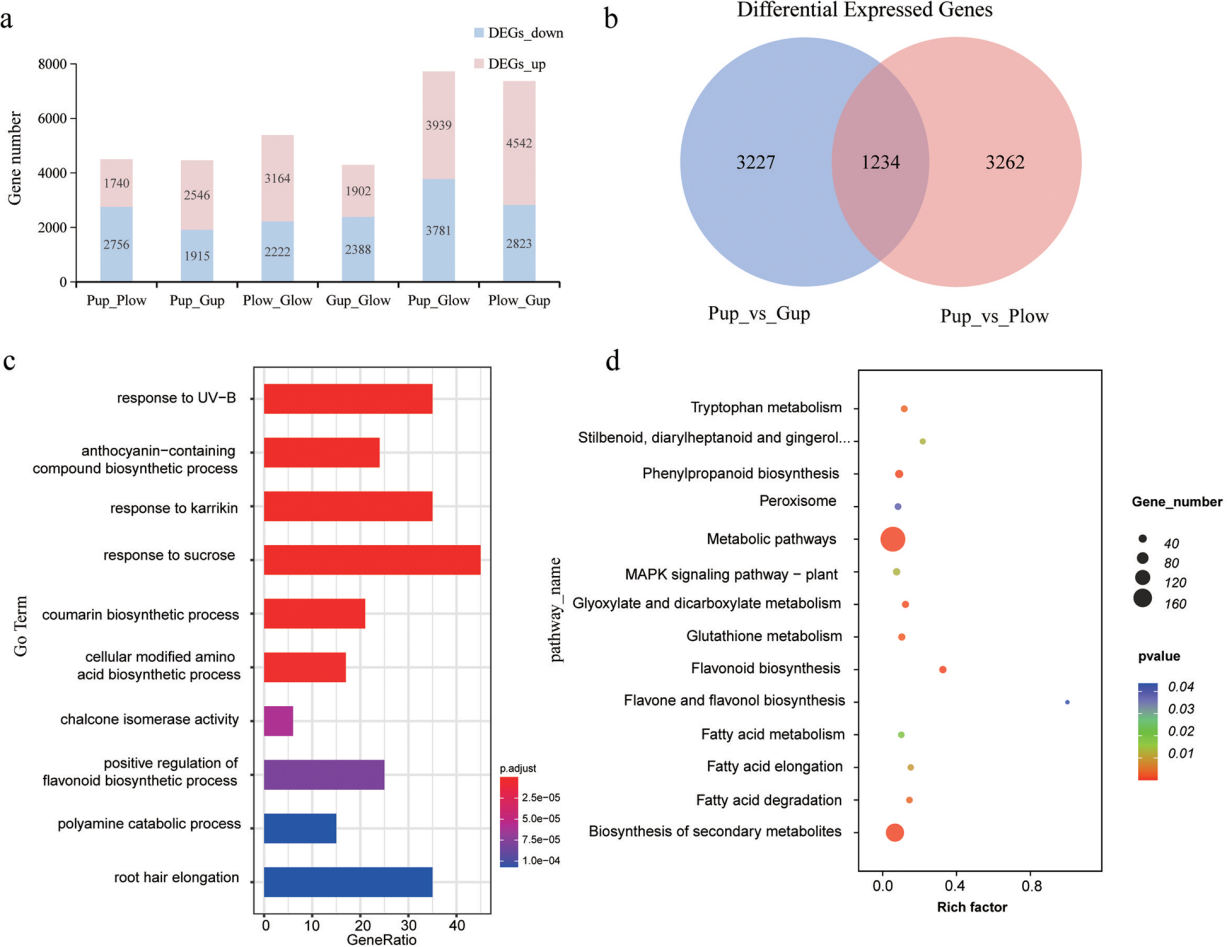
Analysis of differentially expressed genes in transcriptome. **a** The number of DEGs in Pup/Plow, Pup/Gup, Plow/Glow, Gup/Glow, Pup/Glow, Plow/Gup. **b** Venn diagram of DEGs between Pup/Gup and Pup/Plow. **c** GO enrichment analysis of 1234 DEGs based on Pup/Gup and Pup/Plow results, showing the 10 most enriched GO terms. High and low *P* values are indicated in blue and red, respectively. **d** KEGG enrichment analysis of 1234 DEGs based on Pup/Gup and Pup/Plow results, showing the 14 most enriched KEGG terms. High and low P values are indicated in blue and red, respectively

DEG analysis was performed to identify the DEGs in each sample. The transcript levels of the genes were expressed in FPKM values. Four samples were compared to each other, for a total of six groups. In groups Pup_Plow, Pup_Gup, Plow_Glow, and Gup_Glow, about 4290–5386 DEGs were identified. There are more DEGs in Pup_Glow and group Plow_Gup, about 7365–7720 (Fig.3a). The number of DEGs in the upper and lower epidermis of the leaves was similar, suggesting that these genes were expressed in the same location in ZBC or LBC leaves. We also found that the number of DEGs in groups Pup_Glow and Plow_Gup was much higher than that in other groups, which indicates that the expression of genes in the upper and lower epidermis of different color leaves is remarkable. Anyway, the expression of genes in leaves is spatially variable, while the differences become more significant in different materials.

### Functional annotation and classification of DEGs

We screened DEGs in groups Pup_Gup and Pup_Plow to identify which genes were expressed mainly on the upper epidermis of ZBC leaves. This analysis allowed us to further investigate these specific DEGs. Finally, we obtained 1234 genes that were differentially expressed only on the upper epidermis of ZBC leaves (Fig.3b). Then, GO functional enrichment analysis was performed on 1234 DEGs (*p* < 0.05). The results showed that the DEGs were mainly enriched in terms of response to UV-B (GO:0010224), biosynthesis process of anthocyanin-containing compounds (GO:0009718), response to karrikine (GO:0080167), response to sucrose (GO:0009744) and biosynthesis process of coumarin (GO:0009805) (Fig.3c; Table S3).

In addition, we also analyzed the DEGs for KEGG pathway enrichment. Metabolic pathways and biosynthesis of secondary metabolites were the most significant among all KEGG classifications, followed by flavonoid biosynthesis, phenylpropanoid biosynthesis, and glyoxylate and dicarboxylate metabolism (Fig.3d; Table S4).

### Verification of RNA-Seq data by qRT-PCR

To verify the quality of the RNA sequencing results, we selected nine genes involved in the anthocyanins synthesis for verification by quantitative reverse transcription PCR (qRT-PCR). Anthocyanin biosynthetic genes are usually divided into two subgroups: early biosynthetic genes (EBG) and late biosynthetic genes (LBG). EBGs are also flavonoid biosynthesis genes [5]. In our results, the expression levels of both EBGs and LBGs were significantly up-regulated in Pup, such as *CHS*, *CHI*, *DFR*, *ANS*, and so on (Fig. S1). The anthocyanin-regulated genes *MYB111*, *TT8* and *EGL3* were also highly expressed in Pup (Fig. S1). RNA-Seq and qRT-PCR analyses showed similar expression patterns of these genes, indicating the reliability of the transcriptomic data in our study.

### Expression patterns of genes involved in anthocyanin biosynthesis pathway

**Fig. 4:**
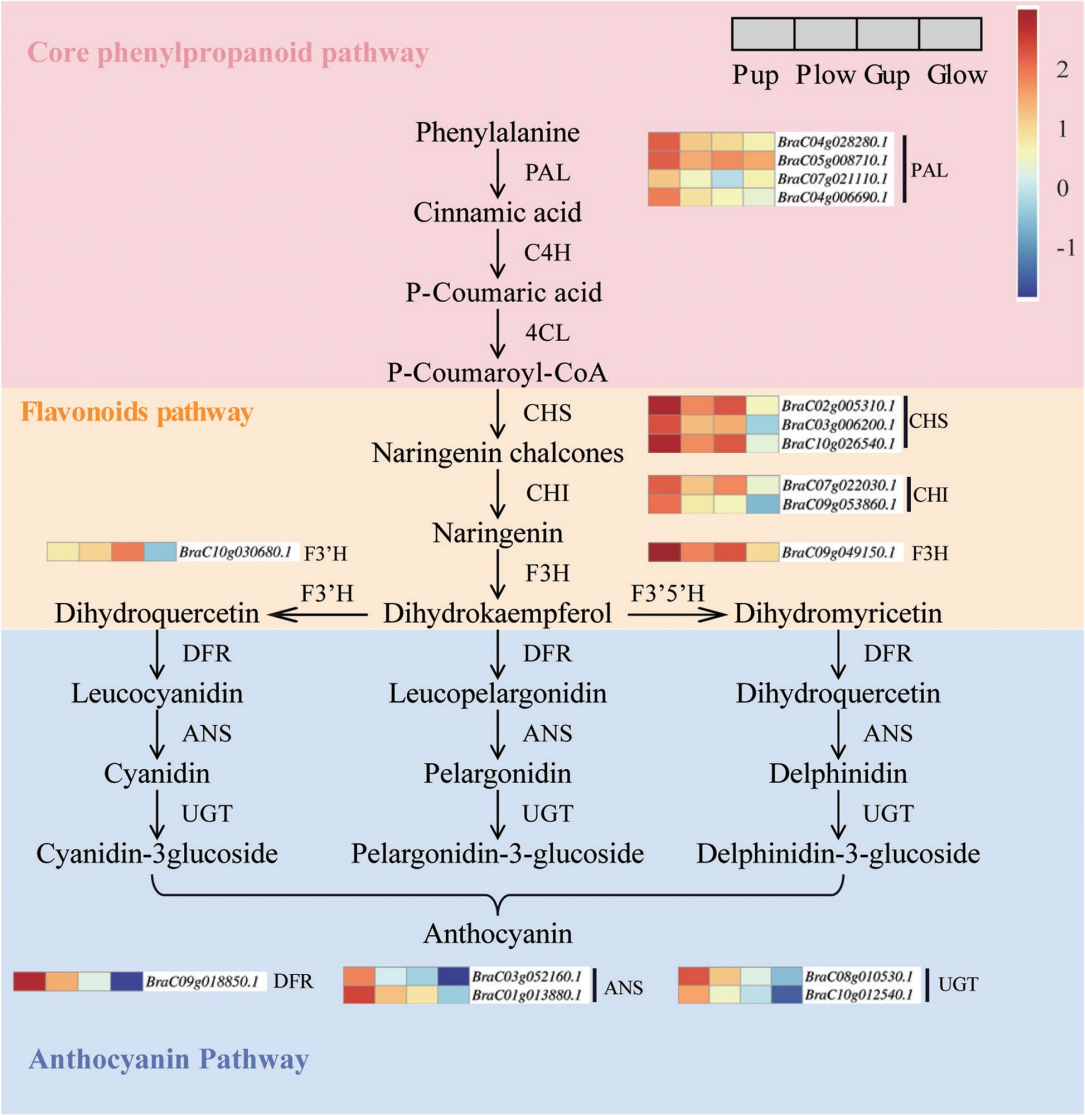
Heat map of gene expression patterns of anthocyanin biosynthetic pathway [45]. Expression patterns are shown on 4 grids Pup, Plow, Gup, Glow. PAL, phenylalanine ammonia lyase; C4H, cinnamate 4-hydroxylase; 4CL, 4-coumarate: CoA ligase; CHS, chalcone synthase; CHI, chalcone isomerase; F3H, flavanone 3-hydroxylase; F3’H, flavanone 3′-hydroxylase; F3’5’H, flavonoid 3′, 5′-hydroxylase; FLS, flavanol synthase; DFR, dihydroflavonol 4-reductase; ANS, anthocyanin synthase; UGT, UDP-glucosyltransferase

To enhance comprehension of the expression patterns of anthocyanin related genes in purple ZBC, we selected genes engaged in the anthocyanin synthesis pathway from 1234 DEGs and constructed an expression heat map. The biosynthetic pathway of phenylacetone involves four genes, *BraC04g028280.1*, *BraC05g008710.1*, *BraC07g021110.1*, *BraC04g006690.1*, all of which are annotated as phenylalanine ammonia lyase (PAL). There are seven genes related to the flavonoid synthesis pathway. *BraC02g005310.1*, *BraC03g006200.1*, *BraC10g026540.1* are annotated as chalcone synthase (CHS), *BraC07g022030.1*, *BraC09g053860.1* as chalcone isomerase (CHI), *BraC09g049150.1* as flavanone 3-hydroxylase (F3H) and *BraC10g030680.1* as flavanone 3′-hydroxylase (F3’H). These genes belong to the EBGs, provide precursor substrates for flavanol and anthocyanin synthesis. The anthocyanin biosynthetic pathway involves five genes, *BraC09g018850.1* are annotated as dihydroflavonol 4-reductase (DFR), *BraC03g052160.1, BraC01g013880.1* as anthocyanidin synthase (ANS), *BraC08g010530.1*, *BraC10g012540.1* as UDP-glucosyltransferase (UGT). The biosynthesis and modification of anthocyanins is controlled by these LBGs (Fig.4). By analyzing the patterns of gene expression, we found that almost all of these anthocyanin-related genes were up-regulated in Pup, consistent with the phenotype of ZBC leaves.

### Expression patterns of BcDFR in ZBC and LBC

Previous studies have shown that DFR, which is the first enzyme in the biosynthetic pathway, exhibits the highest specificity for anthocyanin production and is regulated by multiple transcription factors [31, 32]. In our work, notably, the expression of *BcDFR* (*BraC09g018850*) was most significantly up-regulated in Pup, consistent with the qRT-PCR results (Fig. S1). These findings indicate that *BcDFR* may play a critical role in regulating the specific enrichment of anthocyanins on the upper epidermis of ZBC leaves.

**Fig. 5:**
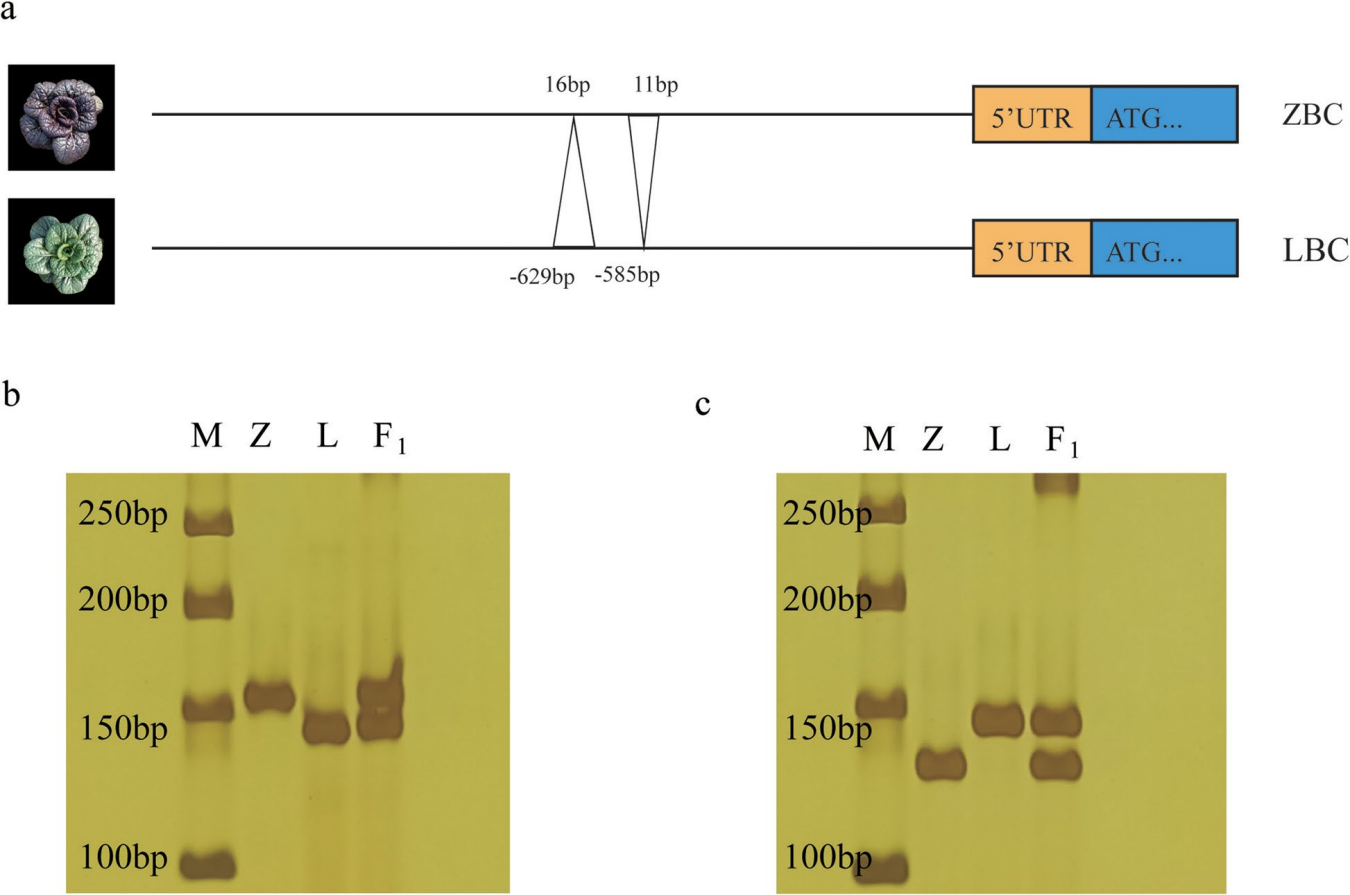
Allelic variation in the 1296 bp promoter region of BcDFR between ZBC and LBC. **a** Schematic representation of allelic variation in the *BcDFR* 1296 bp promoter region between purple ZBC and green LBC. **b** Polyacrylamide gel electrophoresis showing the amplification profile of the deletion marker on ZBC, LBC and F_1_. **c** Polyacrylamide gel electrophoresis showing the amplification profile of the insertion marker on ZBC, LBC and F_1_

To understand why *BcDFR* is highly expressed in Pup but barely expressed in the other three parts, we cloned full-length *BcDFR* from ZBC and LBC*.* The gene sequences of the two cultivars, including exons, introns, and UTR structures, showed a high degree of consistency (Fig.S2a). This suggests that the difference in expression is not caused by variations in gene sequence, prompting us to further analyze the promoter region. Compared with ZBC, *BcDFR* of LBC showed an 11 base pair deletion at − 585 and a 16 base pair insertion at − 629 in the promoter region (Fig. 5a, S2b). Two additional primers were developed based on the specific deletions and insertions sequence of *BcDFR* promoter, to accurately differentiate the genotypes of ZBC, LBC and F_1_ (hybrid generation of ZBC and LBC). In contrast to ZBC, LBC showed 16 bp insertion and 11 bp deletion at these two loci and F_1_ showed heterozygous banding patterns (Fig.5) bc, S3. The insertion and deletion potentially be responsible for the low expression of *BcDFR* in LBC.

### Analysis of regulatory elements and gene structure of DFR

To further understand the evolution of the *DFR* gene, we analyzed the gene structure of it in *Arabidopsis* and six *Brassica species*, including *B. napus*, *B. rapa*, *B. carinata*, *B. nigra*, *B. juncea*, and *B. oleracea.* Eight *DFR* genes are highly conserved, containing six exons and five introns. Domain analysis revealed 10 conserved domains within the *DFR* homologous gene, with only the absence of motif 7 in *BjuB001305* (Fig.S4).

**Fig. 6:**
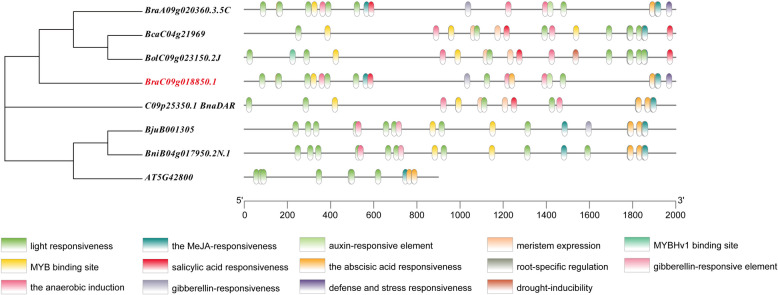
Cis-element analysis in the promoter regions of the DFR genes in Arabidopsis and six Brassica species. Plant CARE software was used to determine the presence of different cis-acting elements, and the different cis-elements are represented in different colored boxes

Cis-elements in the promoter region of the *DFR* gene indicate that these regulatory elements were associated with light responsiveness, MYB binding sites, anaerobic induction, salicylic acid responsiveness, meristem expression and a variety of phytohormone responses (https://bioinformatics.psb.ugent.be/webtools/plantcare/html/). These findings indicate that *DFR* was involved in various aspects of plant growth and development and exerts a significant influence. In addition, there are more binding sites for light responsiveness and anaerobic induction elements in the *DFR* promoter (Fig.6), suggesting that some stress-related genes probably play a critical role in the anthocyanin biosynthesis of plants. In addition, we also found many MYB binding sites on *DFR* promoter, suggesting that MYB transcription factors may act as upstream of *DFR* and then regulate its expression.

### Prediction of BcDFR-mediated protein-protein interaction networks

To better understand the function of *BcDFR* in the anthocyanin synthesis pathway, we used STRING to predict the protein interaction network of BcDFR (Fig.S5). The results showed that BcDFR directly interacted with C4H, CHS, CHI, F3’H, FLS, UGT75C1, and TT8 proteins, while it also indirectly interacted with ANS, F3H, PAL, MYB111, and MYBL2. Interestingly, MYBL111, TT8, together with MYBL2, directly or indirectly affect catalysis proteins for the whole anthocyanin biosynthesis network. Further, RNA-Seq analysis showed that *BcMYBL111*, *BcTT8*, and *BcMYBL2* were substantially up-regulated in Pup (Fig.S6). The specific expression of them also impacts the elevated expression of *BcDFR* in Pup, which may result in the anthocyanins only accumulating in Pup.

### Transient expression of *BcDFR* promotes anthocyanin accumulation

**Fig. 7:**
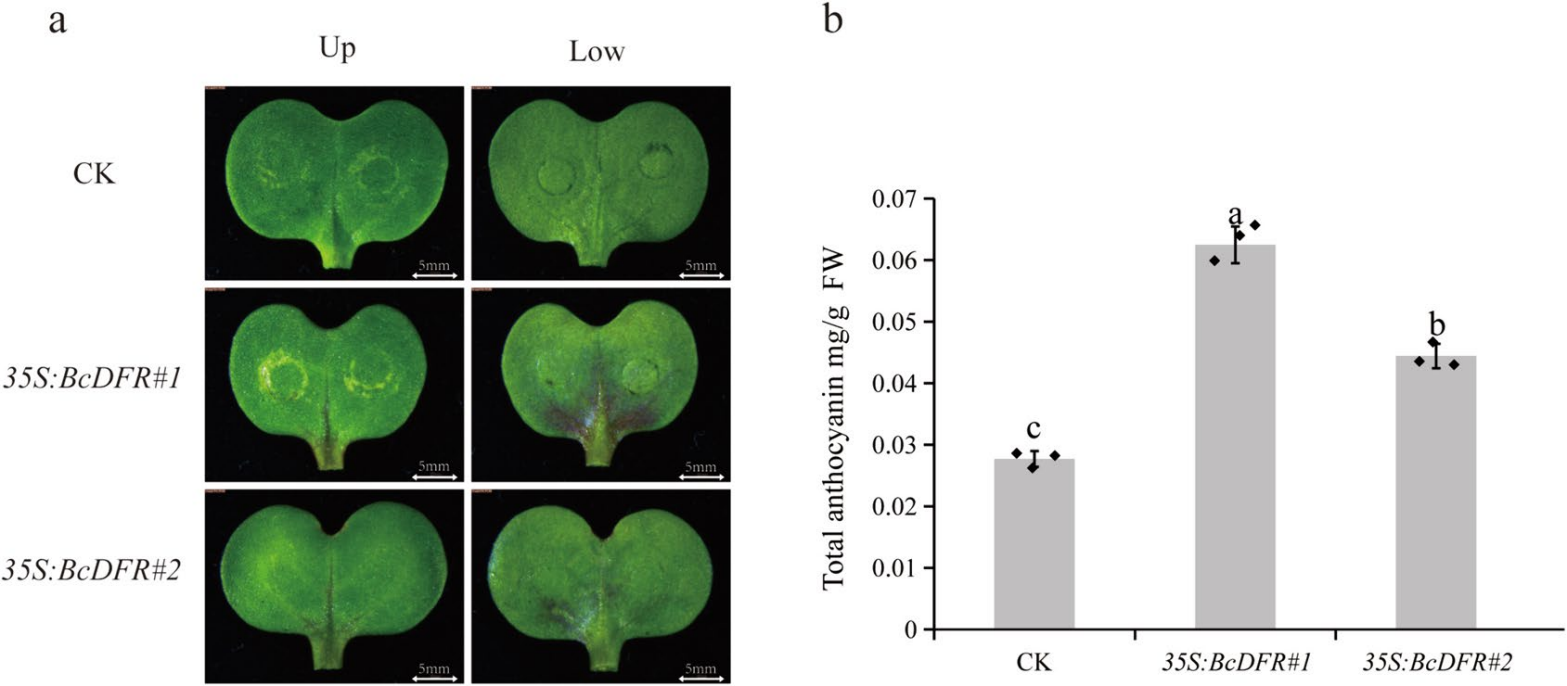
Phenotype and anthocyanin content of BcDFR transient expression in LBC cotyledon. **a** Phenotypes of upper and lower epidermis LBC cotyledons after injection for 7 days. **b** Total anthocyanin content of CK and *35S: BcDFR* LBC cotyledons. CK, empty vector controlling; *35S: BcDFR*, *BcDFR* overexpression. The data are from three independently repeated experiments. Error bars represent±SD. Different letters denote significant differences (*p* < 0.05)

To further study *BcDFR* function in anthocyanin accumulation, we inject *35S: BcDFR* into LBC cotyledon for the transient expression assay. We used the empty vector as a control. Compared to the control, the lower epidermis of green cotyledons injected with *35S: BcDFR* showed anthocyanin accumulation (Fig.7a). The total anthocyanin content of the control was 0.028 mg/g, while that of the *35S: BcDFR* cotyledons was 0.044–0.063 mg/g (Fig.7b). This result indicates that *BcDFR* expression promotes anthocyanin synthesis and accumulation.

## Discussion

Non-heading Chinese cabbage [*Brassica campestris* (syn. *Brassica rapa*) ssp. *chinensis*] is a popular leafy vegetable. The accumulation of anthocyanins gives the leaves a purple color and also a higher nutritional value. In this study, we used laser capture frozen section method (LCM) to divide purple ZBC and green LBC leaves into upper and lower epidermis (Pup, Plow, Gup, Glow). RNA-seq analysis combined with functional verification indicates that *BcDFR* is a key gene regulating anthocyanin accumulation in non-heading Chinese cabbage leaves.

### *DFR* is essential for anthocyanin synthesis and accumulation

Many enzymes are involved in anthocyanin biosynthesis [33]. DFR plays an essential role as the first catalytic protein in the anthocyanin biosynthetic pathway. Under the help of ANS, UFGT, and NADPH, DFR selectively catalyzes dihydroquercetin, dihydrokaempferol, and dihydromyricetin formation of cyanidin-3glucoside, pelargonidin-3-glucoside, and delphinidin-3-glucoside [34]. O’Reilly et al. [35] used the translocon labeling method to first isolate the *DFR* gene from maize and later obtain it from *Petunia* [36], and other species such as *Gerbera* [37], *Ginkgo* [38], and *Scutellaria* [39]. DFR plays a crucial role in plant coloration. Previous studies have shown that *BoDFR1* is a candidate gene for controlling red or purple leaves in *Kale* [39]. The mutations in *BoDFR1* render it non-functional, disrupting anthocyanin synthesis and accumulation [40]. In our study, we combined RNA-seq and qRT-PCR to identify *BcDFR*, which is the most up-regulated gene in the upper epidermis of purple leaves. Sequence analysis of *BcDFR* in ZBC and LBC showed a deletion and an insertion fragment in the promoter region. It remains to be investigated how these variants affect *BcDFR* expression.

### Anthocyanin specific accumulation of non-heading Chinese cabbage leaves

Anthocyanin is a key factor in determining the purple color phenotype of leaves. The color is determined by the accumulation of anthocyanin, which is primarily regulated by genes in the anthocyanin pathway [41]. These genes have been relatively well studied, and we have a relatively accurate understanding of their function and expression patterns [42, 43]. In this work, the anthocyanins of purple ZBC only accumulated on the upper epidermis of the leaves, while the lower epidermis were green (Fig.1) de. Traditional quantitative detection often targets the whole leaf and cannot differentiate the differences between the upper and lower epidermis of the leaf. Therefore, in this study, we used the LCM method for the first time to divide the leaves into upper and lower parts. Combined with transcriptome sequencing, it provides more comprehensive and accurate gene expression data for in-depth research on the specific accumulation of anthocyanins.

To determine whether anthocyanin enrichment is caused by the specific expression of the genes, we analyzed the gene expression of the anthocyanin synthesis pathway. We found that almost all of these anthocyanin-related genes were up regulated in Pup (Fig.4, S1), consistent with the phenotype of ZBC leaves. The predicted protein-protein interaction network of BcDFR suggested that BcDFR has the ability to interact with most all genes associated with the anthocyanin biosynthetic pathway, in addition to its association with MYB111, MYBL2, and TT8 (Fig.S5). Further, RNA-Seq analysis showed that *MYBL111*, *TT8*, and *MYBL2* were substantially up-regulated in Pup (Fig.S6). Thus, specific expression of anthocyanin synthesis pathways and regulatory genes may be the reason for specific accumulation of anthocyanins. Interestingly, *MYBL2* had been reported as a negatively regulated gene for anthocyanins [44], but was upregulated in Pup. These results need to be further explored.

In conclusion, we used LCM method to study DEGs that are specifically expressed in the upper epidermis of purple non-heading Chinese cabbage leaves. Finally, our study identified *BcDFR*, a key gene in the anthocyanin synthesis pathway. Our study provides new insights into the functional analysis and transcriptional regulatory network of anthocyanin-related genes in purple non-heading Chinese cabbage.

### Supplementary Information

**Supplementary Material 1.**

**Supplementary Material 2.**

## Data Availability

The RNA-Seq datasets are available in the Sequence Read Archive of National Center for Biotechnology Information. (https://www.ncbi.nlm.nih.gov/bioproject/PRJNA1063459).

## Abbreviations

LCM: Laser capture frozen section method
ZBC: Purple non-heading Chinese cabbage
LBC: Green non-heading Chinese cabbage
Pup: The Purple upper epidermis
Plow: The purple lower epidermis
Gup: The Green upper epidermis
Glow: The green lower epidermis
DEG: Differentially expressed gene
LTZ: Low temperature treated purple non-heading Chinese cabbage
TFs: Transcription factors
EBGs: Anthocyanin early biosynthetic genes
LBGs: Anthocyanin late biosynthetic genes
