# Supplementary material for: Laser capture microdissection transcriptome (LCM RNA-seq) reveals BcDFR is a key gene in anthocyanin synthesis of non-heading Chinese cabbage
Source: BMC Genomics. 2024 Apr 29;25:425. doi: 10.1186/s12864-024-10341-y (PMC11059580; doi:10.1186/s12864-024-10341-y)
Supplement: Supplementary file 1 — Supplementary Material 1. [file 12864_2024_10341_MOESM1_ESM.pdf]

Laser capture microdissection transcriptome (LCM RNA-seq) reveals *BcDFR* is a key gene in anthocyanin synthesis of Non-heading Chinese cabbage

Qian Zhou[1], Xinfeng Xu[1], Mengjie Li[1], Xiaoxue Yang[1], Meiyun Wang[1], Ying Li[1], Xilin Hou[1,2*] and Tongkun Liu[1*]

[1]State Key Laboratory of Crop Genetics & Germplasm Enhancement, Key Laboratory of Biology and Genetic Improvement of Horticultural Crops (East China), Ministry of Agriculture and Rural Affairs of China, Engineering Research Center of Germplasm Enhancement and Utilization of Horticultural Crops, Ministry of Education of China, Nanjing Agricultural University, Nanjing 210095, China

[2]Nanjing Suman Plasma Engineering Research Institute, Nanjing Agricultural University, Nanjing 210095, China

*Corresponding authors. E-mail: hxl@njau.edu.cn; liutk@njau.edu.cn

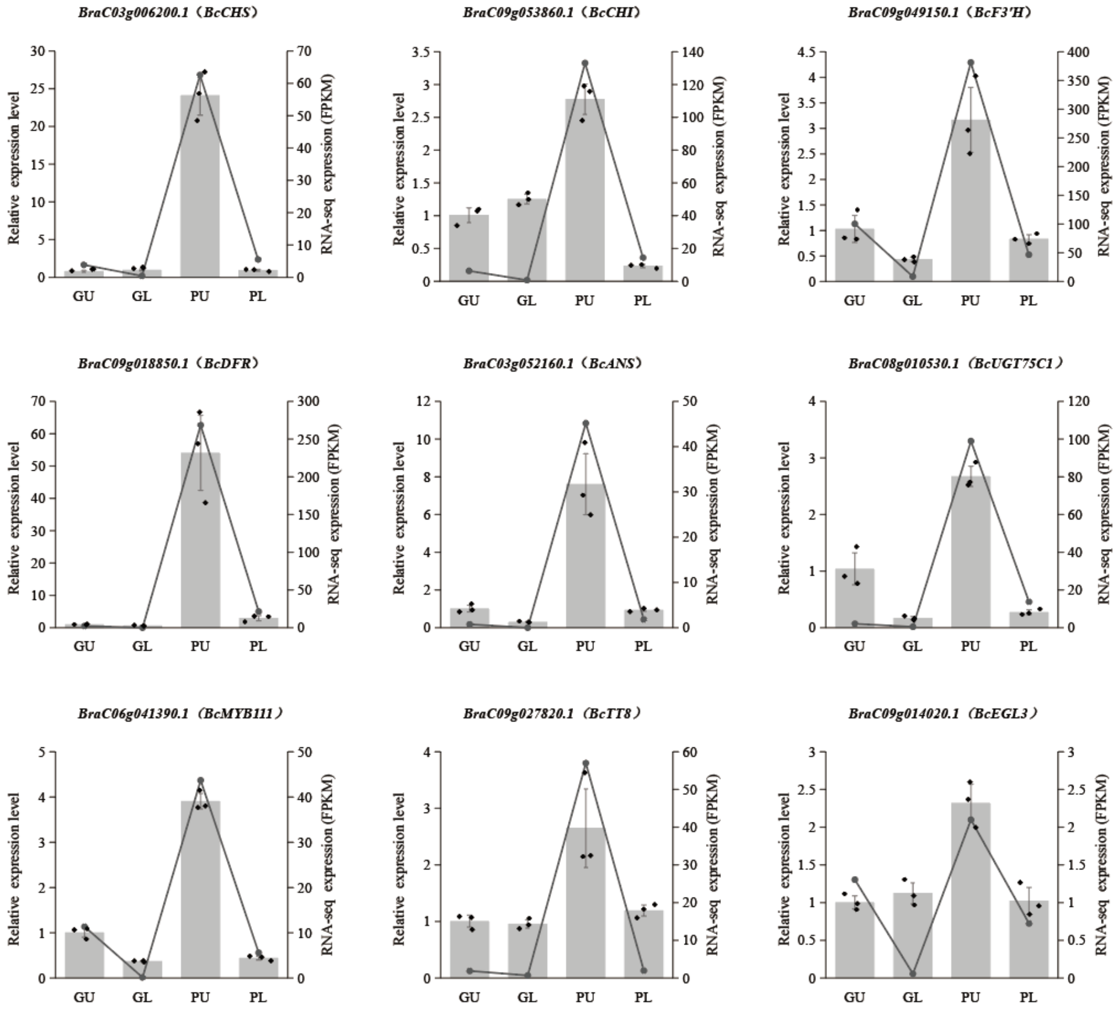

Fig. S1 Relative expression levels of genes involved in anthocyanin biosynthesis. Bar and line graphs represent the qRT-PCR and RNA-seq data, respectively. Scatter plots represent individual data values. Data are presented as mean ± SD of three biological replicates.

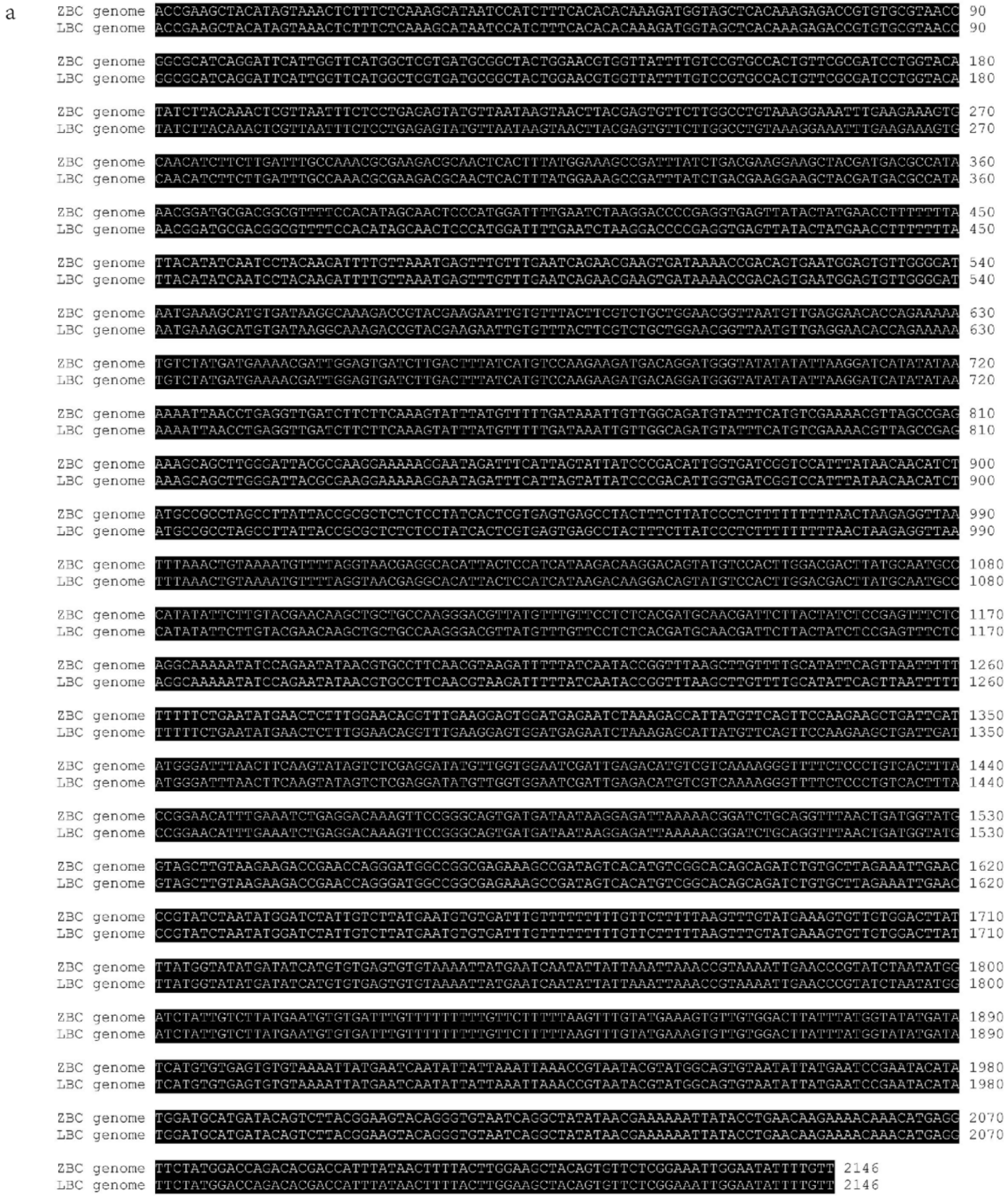

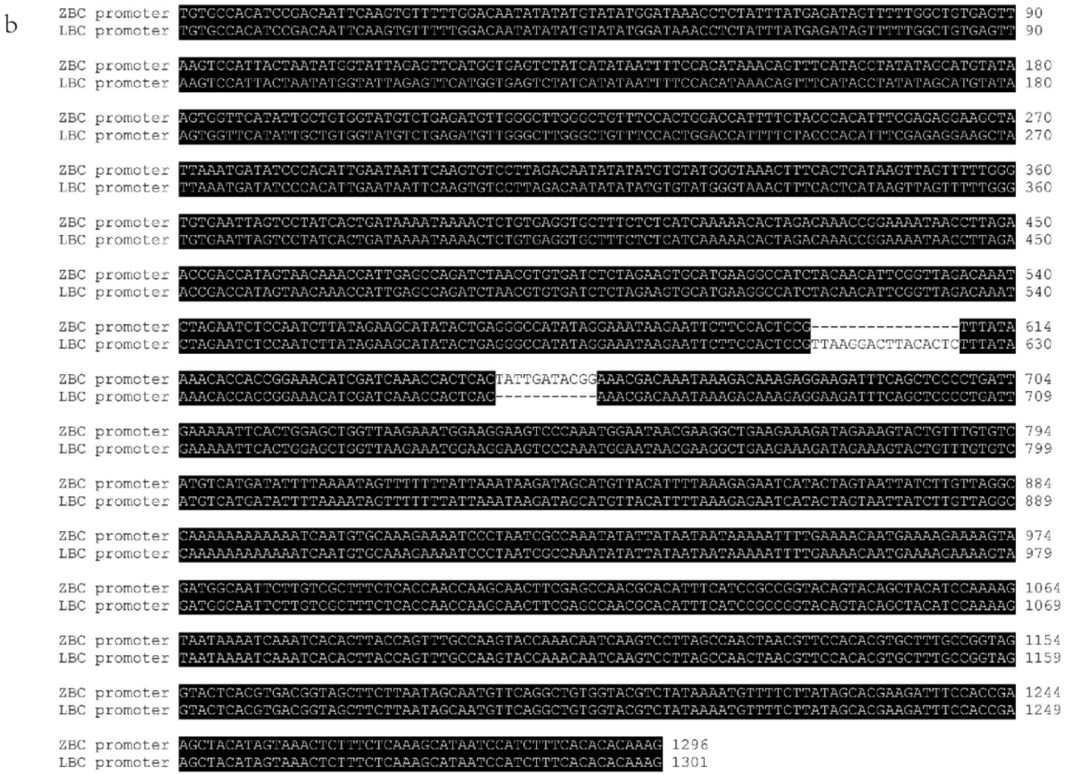

Fig.S2 Gene and promoter sequence comparison results of *BcDFR* in ZBC，LBC. (a) Results of the 2146 bp full length genome sequence comparison. (b) Results of the 1296 bp promoter sequence comparison.

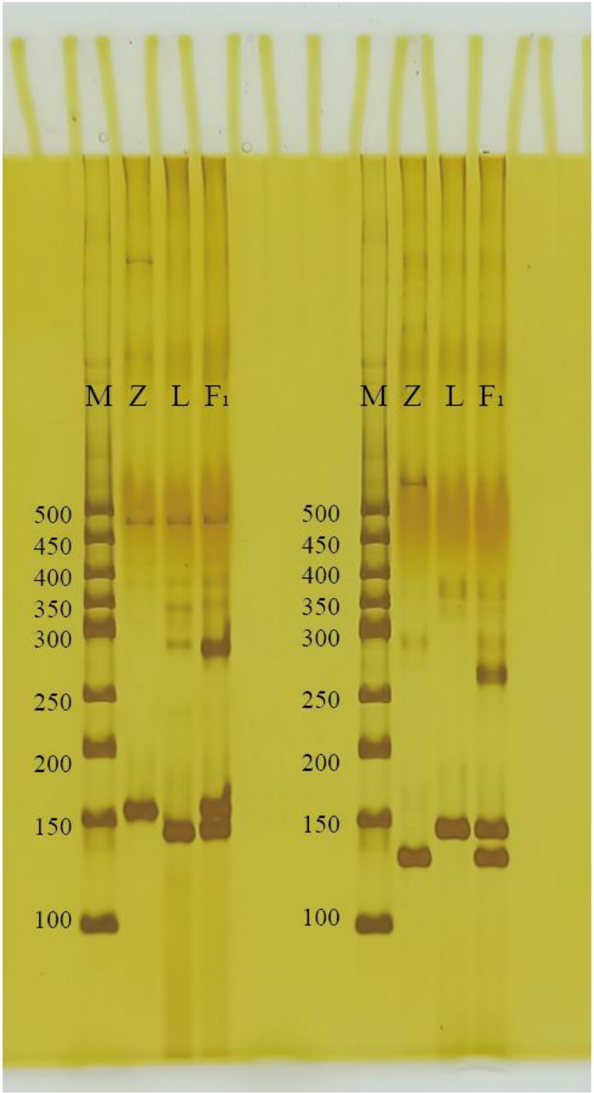

Fig.S3 Full-length polyacrylamide gel electrophoresis of amplification of insertion and deletion markers on ZBC, LBC and F_1.

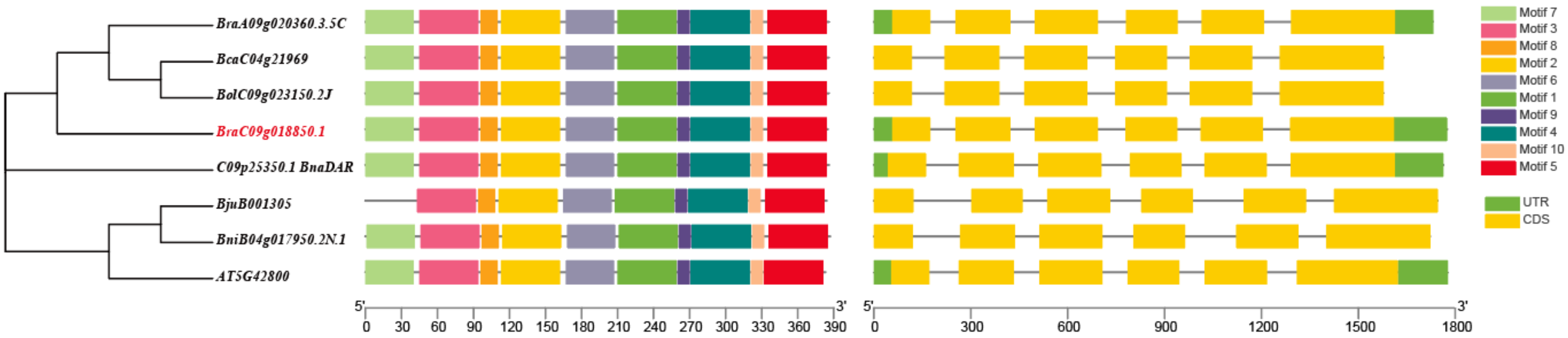

Fig.S4 Evolution, gene structure and conserved domain analysis of DFR homologs in Arabidopsis and six *Brassica species*.

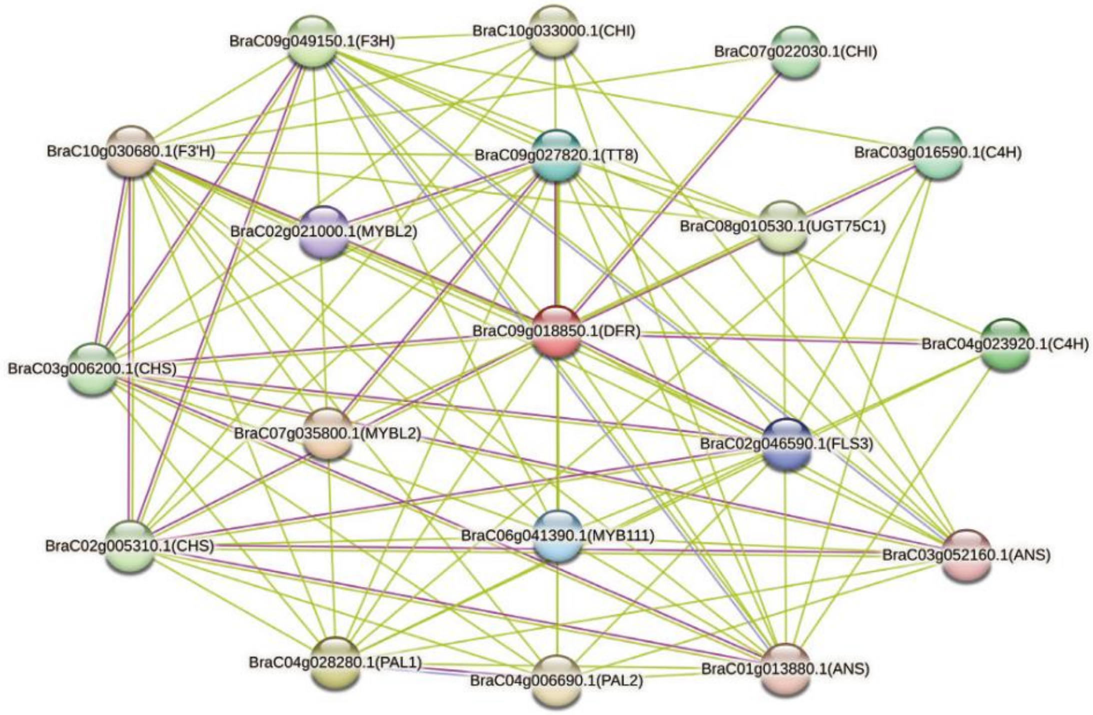

Fig.S5 Protein-protein interaction network between *BcDFR* and anthocyanin-related DEGs.

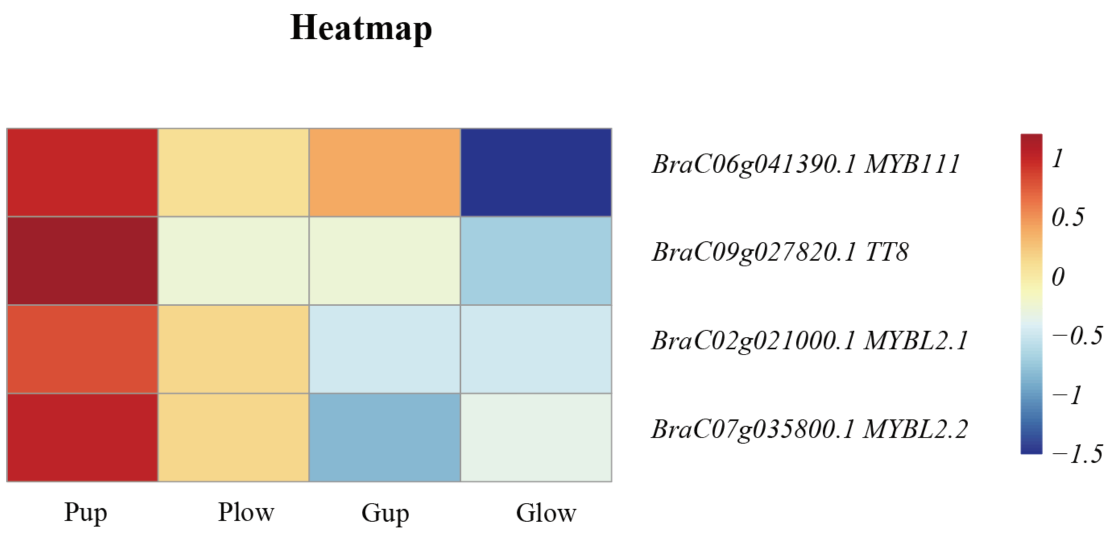

Fig.S6 Heat map of gene expression patterns of *BcMYB111*, *BcTT8* and *BcMYBL2*.
